# The Enigma of Soil Animal Species Diversity Revisited: The Role of Small-Scale Heterogeneity

**DOI:** 10.1371/journal.pone.0011567

**Published:** 2010-07-13

**Authors:** Uffe N. Nielsen, Graham H. R. Osler, Colin D. Campbell, Roy Neilson, David F. R. P. Burslem, René van der Wal

**Affiliations:** 1 The Macaulay Institute, Aberdeen, United Kingdom; 2 School of Biological Sciences, University of Aberdeen, Aberdeen, United Kingdom; 3 Aberdeen Centre for Environmental Sustainability, University of Aberdeen, Aberdeen, United Kingdom; 4 Department of Soil and Environment, Swedish University of Agricultural Sciences, Uppsala, Sweden; 5 Scottish Crop Research Institute, Dundee, United Kingdom; Dalhousie University, Canada

## Abstract

**Background:**

“The enigma of soil animal species diversity” was the title of a popular article by J. M. Anderson published in 1975. In that paper, Anderson provided insights on the great richness of species found in soils, but emphasized that the mechanisms contributing to the high species richness belowground were largely unknown. Yet, exploration of the mechanisms driving species richness has focused, almost exclusively, on above-ground plant and animal communities, and nearly 35 years later we have several new hypotheses but are not much closer to revealing why soils are so rich in species. One persistent but untested hypothesis is that species richness is promoted by small-scale environmental heterogeneity.

**Methodology/Principal Findings:**

To test this hypothesis we manipulated small-scale heterogeneity in soil properties in a one-year field experiment and investigated the impacts on the richness of soil fauna and evenness of the microbial communities. We found that heterogeneity substantially increased the species richness of oribatid mites, collembolans and nematodes, whereas heterogeneity had no direct influence on the evenness of either the fungal, bacterial or archaeal communities or on species richness of the large and mobile mesostigmatid mites. These results suggest that the heterogeneity-species richness relationship is scale dependent.

**Conclusions:**

Our results provide direct evidence for the hypothesis that small-scale heterogeneity in soils increase species richness of intermediate-sized soil fauna. The concordance of mechanisms between above and belowground communities suggests that the relationship between environmental heterogeneity and species richness may be a general property of ecological communities.

## Introduction

The great diversity of soil faunal communities was recognised many decades ago [1], [2]. Since then, the rapid development of molecular methods has revealed an even greater richness of microbes, with as many as 10^4^–10^6^ bacterial operational taxonomical units found in a single gram of soil [3]–[5]. This has led some authors to suggest that a large proportion of species on Earth are found in soils [6]. Yet, the exploration of the mechanisms underlying observed patterns of species richness has, to a great extent, been limited to above-ground terrestrial and aquatic plant and animal communities [7]. Hence, the mechanisms underlying the high species richness of below-ground communities remains to be understood fully [8].

Although there are major differences between above and belowground systems, it has been proposed that some of the mechanisms underlying patterns of species richness aboveground may also be important belowground [9]. For above-ground terrestrial and aquatic systems, it is widely accepted that environmental heterogeneity has a positive influence on species richness [10]. At large scales (e.g. regions or landscapes) species richness increases with the number of habitats occurring within the area being surveyed [11]. Similarly, there is increasing evidence for equivalent positive relationships at smaller spatial scales (e.g. within habitats) for plants [12]–[14] and aquatic invertebrates [15]–[17], with some evidence also for soil fauna. It has been shown, for example, that the diversity of soil mites increases with microhabitat diversity within sites [18]–[19], and that the species richness of both soil mites and nematodes increases with the complexity and heterogeneity of the litter layer [20]–[24]. This does not, however, explain the great species richness observed within small volumes of soil. One of the main characteristics of soils is their high heterogeneity at scales much smaller than those considered above-ground [25]. Hence, it has been hypothesised repeatedly that the great species richness observed in small quantities of soil is related to the high heterogeneity found at very fine scales within the soil [8], [26]–[28]. Due to the intricate nature of soils only a few attempts have been made to explain species richness at such spatial scales, and none of these have tested the importance of small-scale heterogeneity directly.

**Figure 1:**
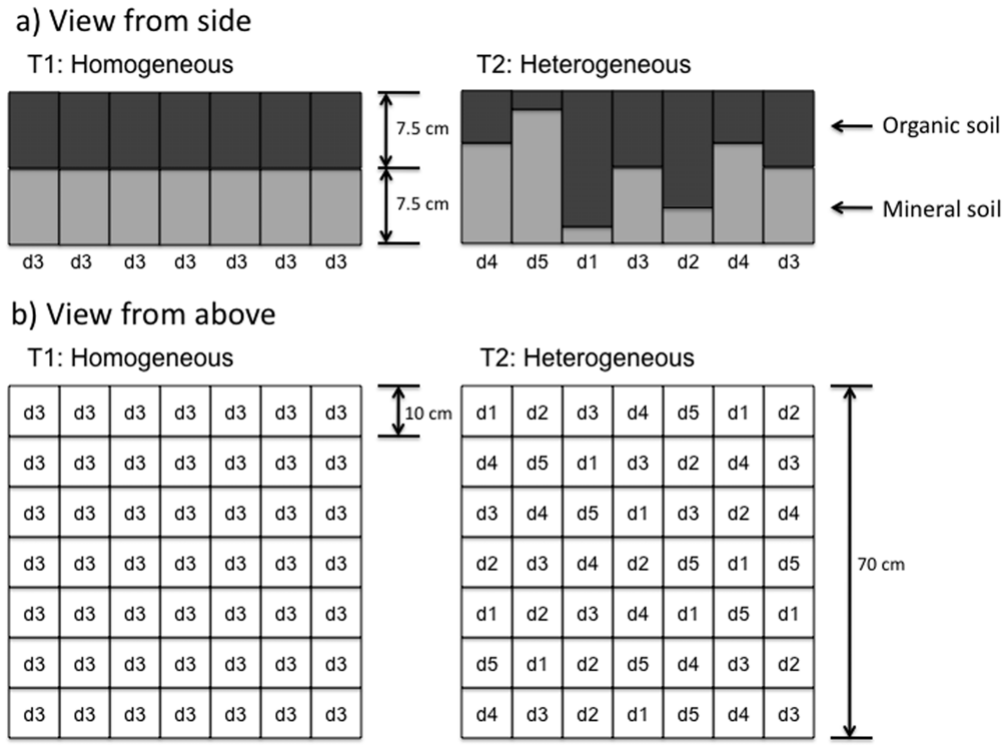
Schematic presentation of the experimental treatments. a) View from the side to show the organic layer depths for both treatments representing either homogeneous or heterogeneous environment. The homogeneous treatment had the same organic (O) horizon depth (7.5 cm) throughout the box whilst the heterogeneous treatment had a mix of 5 different O-horizon depths ranging from a deep (12 cm, d1) to a shallow (3 cm, d5) O-horizon in steps of 2.25 cm increase in depth. b) View from above to show the distribution of the depths throughout each replicate. In the homogeneous treatments three cells (7.5 cm, d3) were sampled to make up one composite sample for each biotic group. In the heterogeneous treatment three cells with the same depth as the homogeneous treatment (d3) were sampled to make up one composite sample (single depth sample), and another composite sample was collected by sampling each of a cell with d1, d3 and d5 (mixed depth sample).

To redress this, we conducted an *in situ* manipulation of the physical properties of a soil by varying the thickness of the organic horizon at small scales to create environments with either low or high heterogeneity in depth of the organic soil horizon (Fig. 1). This manipulation was designed to influence soil moisture regimes and thereby a wide range of soil properties and processes [29]. We excluded plants from our experimental design as variation in plant community composition could influence soil communities. Hence, our manipulation of small-scale heterogeneity is equivalent to the natural variation found due to soil topology or the physical impact of a large root or a rock on soil structure and soil properties. We investigated the influence of this heterogeneity on species richness of soil mites, springtails and nematodes, and the evenness of some components of the microbial communities, as these groups represent a large, although not exhaustive, part of the soil food web.

## Results

We first explored the influence of heterogeneity on the abundance of soil animals. We took three sub-samples (5×5 cm and 6 cm depth) from cells with an organic horizon thickness of 7.5 cm in all replicates of either treatment. From these samples we extracted mesostigmatid and oribatid mites, collembolans and nematodes to cover a wide range of soil organisms. Mean abundance appeared higher for all four groups in the heterogeneous soil environment, although this difference was only statistically significant for nematodes (Table 1). This could be due to any number of factors including, but not limited to, biotic interactions such as a decrease in interspecific competition or changes in the strength of mutualistic or facilitative relationships.

**Table 1:** Summary of abundance data (mean ± s.e., n = 8) of mesostigmatid and oribatid mites, collembolans and nematodes (ind. ×10^3^ m^−2^) in the top 0–6 cm of the organic horizon in treatments representing either homogeneous or heterogeneous soil environment.

	Homogeneous	Heterogeneous	*F*_3,15_	P
Mesostigmata	8.5±1.2	10.4±0.9	1.48	>0.20
Oribatida	12.6±1.0	19.2±3.0	4.51	>0.05
Collembola	91.1±19.1	118.4±15.7	1.38	>0.25
Nematodes	**187.1±19.8**	**269.7±37.0**	**6.34**	**<0.05**

*F* statistic and associated significance level (one-way ANOVA with blocks; bold if significant) are presented.

**Figure 2:**
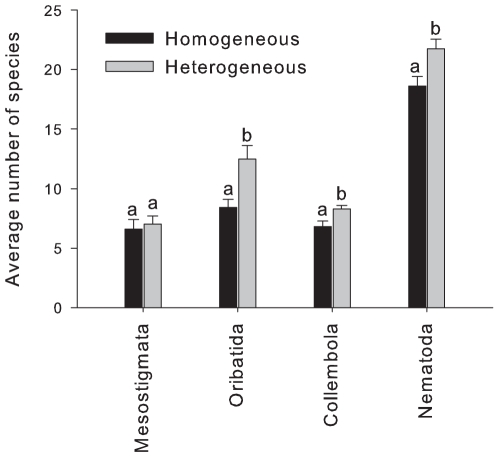
Average species richness (mean ± s.e., n = 8) at 0–6 cm depth found in the two treatments for the biotic groups sampled. Letters indicate significant within-group differences between treatments (one-way ANOVA with blocks).

We then compared the species richness of soil fauna in the soil cores used to determine differences in abundance between treatments (i.e. all 7.5 cm organic horizon thickness cores). In these cores the species richness of oribatid mites, collembolans and nematodes was 22–49% greater in the heterogeneous treatment than in the homogeneous treatment, whereas species richness of the mesostigmatid mites was unaffected (Fig. 2). We also determined the evenness (expressed as Simpson's evenness measure E_1/D_) of the microbial communities using Terminal Restriction Fragment Length Polymorphism for these samples, as this measure is more closely related to species richness of the soil microbial community than the number of fragments found in a sample [30]. Evenness did not differ significantly between homogeneous and heterogeneous treatments for fungi, bacteria or archaea (p>0.05 in all cases).

**Figure 3:**
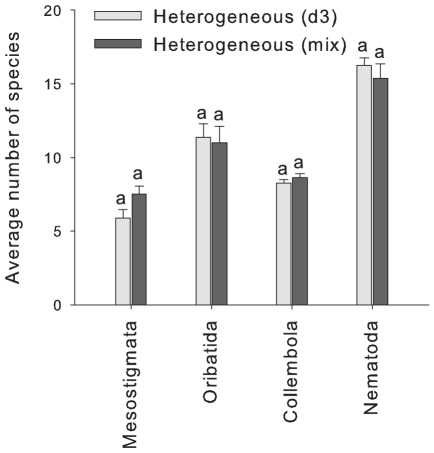
Average species richness (mean ± s.e., n = 8) at 0–3 cm depth found in the heterogeneous treatment for the biotic groups sampled. Both sampling regimes used in the heterogeneous treatment are presented: d3 represents the same depth as sampled in the homogeneous treatment, whereas mix represents pooled samples collected across shallow (3 cm), medium (7.5 cm) and deep (12 cm) organic horizons.

We expected differences in affinity of various groups of soil biota to organic soil of certain depths due to associated differences in soil moisture (i.e. between deep, medium and shallow organic horizons, with deep horizons being more moist than shallow horizons). Hence, we used another sampling regime to determine if certain species within each group were unique to specific organic horizon depths in the heterogeneous treatment only. We explored whether the species richness of soil fauna and microbial community evenness within the heterogeneous treatment was similar when sampling only one organic horizon thickness (7.5 cm only, i.e. the single depth sample as explained in Fig. 1) compared with sampling multiple organic horizon thicknesses (3, 7.5 and 12 cm combined, i.e. the mixed depth sample) from within the same treatment. If some species would occur only in specific organic horizon thicknesses, then the species richness or evenness should be greater in the mixed depth sample. However, we found no apparent difference between the two sampling regimes in the species richness of mites, collembolans or nematodes (Fig. 3) or in the evenness of the microbial communities. This suggests that heterogeneity allowed the co-existence of more species of oribatid mites, collembolans and nematodes, but that the species were not restricted to specific organic horizon thicknesses.

## Discussion

Our results demonstrate clearly that small-scale heterogeneity in soils influences species richness of intermediate-sized soil fauna. Although the diversity of soil mites within a site has been related to the diversity of microhabitats [18]–[19], and the species richness of soil mites and nematodes to litter complexity and heterogeneity [20]–[24], this is the first experimental demonstration that species richness belowground increases in response to an increase in small-scale heterogeneity within the soil itself. A similar relationship has been shown between small-scale environmental heterogeneity and species richness of plants [12]–[14] and aquatic invertebrates [15]–[17]. Therefore, our results support the notion that similar mechanisms underlie patterns of species richness in aquatic, and above and belowground terrestrial ecosystems. This concordance of mechanisms across scales and communities suggests that the relationship between environmental heterogeneity and species richness may be a general property of ecological communities.

The species richness found in this experiment (19 and 32 species of mesostigmatid and oribatid mites, respectively, 13 species of collembolans and 35 species of nematodes) is comparable to other studies [31], [32]. This suggests that species of all groups, including non-opportunist species, had been able to colonize the treatments. Although the overall abundance of nematodes was significantly different between treatments, the number of identified individuals was similar between treatments, and any treatment effect on species richness could therefore not be due to sampling effects.

The mesostigmatid mites did not show a response to the increase in heterogeneity provided by our manipulations of the O-horizon thickness. Most mesostigmatid mites are relatively large (up to 2 mm), mobile and voracious predators. Although some species show microhabitat preferences [33], their size and mobility reduce the likelihood that this group would show a strong relationship with heterogeneity in soil properties at the small scales used in this experiment, as they could disperse freely across the treatments. In contrast, the species richness of collembolans, oribatid mites and nematodes was greater in the heterogeneous than in the homogeneous treatment, which suggests that these groups respond to heterogeneity at the scale of the treatments. However, the similarity in species richness of these groups in single- and mixed-organic horizon depth samples of the heterogeneous treatment suggests that these species disperse at scales >10 cm. It appears that, although the species richness increased due to an increase in microhabitat availability created by the additional organic horizon depths, all species migrated between the compartments with different organic horizon depths within the heterogeneous treatment.

We found no measurable effect of heterogeneity on the evenness of the microbial communities. While this may suggest that their species richness did not respond to heterogeneity at this scale it might also be that we have not measured the true extent of their diversity using TRFLP. Spatial isolation of individuals on soil aggregates or in soil pores may increase species richness of soil microorganisms due to a decrease in competition [34]–[36]. Hence, spatial isolation at micro-scales within soils (10^−3^–10^−1^ cm) may have a stronger influence on the species richness of microorganisms than the small-scale (10^0^–10^1^ cm) heterogeneity imposed by our manipulations. However, given the evidence found for other soil biota presented here we predict a similar relationship for microbes will emerge when measured at the appropriate scale and/or by techniques that cover a greater proportion of the diversity.

The influence of heterogeneity on species richness was substantially greater for oribatid mites (49% increase) than for collembolans (22% increase). This difference may be related to their feeding preferences and choice of microhabitats. Even though some oribatid mites occupy the litter layer, many species are widely distributed throughout the organic horizon. Similarly, many other species of soil animals show strong preferences towards specific soil horizons [37] or to particular physico-chemical environments within the soil [38]–[39]. In contrast, the collembolans, which feed predominantly on fungal hyphae or decaying organic matter, may be more closely associated with the litter layer [40]. Hence, the similar litter type used for both treatments may have limited the response of species richness of collembolans to heterogeneity in soil properties.

We have found strong evidence across several groups of soil organisms for an influence of environmental heterogeneity on species richness, notably that the majority of the mesofauna were governed by heterogeneity at our ‘intermediate’ scale. The absence of a similar detectable relationship for the smallest and the largest organisms examined (i.e. the microbes and the mesostigmatid mites) is interesting. It appeared that the mesostigmatid mites are sufficiently mobile that all species occur by chance in both treatments, while the less mobile component of the mesofauna, i.e. the oribatid mites, collembolans and nematodes, were sufficiently dispersal-limited to allow biotic interactions within the treatments to influence species richness at this scale. Our experiment has therefore highlighted the different scales at which heterogeneity might affect soil community assembly. If we extend these principles we can suggest this is likely to be true for microbial richness if they too are dispersal limited, and if we can obtain data in the future at the appropriate scale and using techniques that capture the true extent of their diversity. As species-rich communities of soil organisms contribute to the provision of ecosystem services [41], it is important to know which factors promote their species richness. We conclude that heterogeneity in the topography and structure of soils at small spatial scales, whether created through physical or biological processes, may be of particular importance for promoting species richness of soil biota, although the response is scale-dependent and varies according to organism size and behaviour. These results are consistent with the interpretation that niche partitioning plays an important role in community assembly. The corollary of this is that any activity that homogenizes soils, such as cultivation, may reduce soil biodiversity, which could alter the ecosystem services provided by the soil biota.

## Materials and Methods

### Experimental design

The experiment was established in April 2007 (spring) within a birch woodland at the Centre for Ecology and Hydrology, Banchory, UK (57°04′N, 2°32′W). We chose birch woodland as we expected there to be a relatively large species pool from which colonization could occur. We manipulated the depth of the O-horizon to create two treatments with either low or high heterogeneity in soil properties (Fig. 1) with eight replicates of each treatment. Hence, 16 holes, each measuring 70×70 cm and 15 cm deep, were dug within the birch woodland. In each of the holes we created 49 ‘cells’ measuring 10×10 cm and 12 cm deep using a 1030 µm nylon mesh (dimension between the midpoint of adjacent sides of the opening) with an open area of 57%. The nylon mesh was rigid enough to prevent soil from different compartments mixing during establishment and for the duration of the experiment. The upper 3 cm was not divided by mesh to allow free movement of larger soil fauna, i.e. earthworms, enchytraeids and some soil dwelling microarthropods. In the homogeneous treatment (T1) we added a mineral soil (sterilised loam) to each cell up to 7.5 cm from the bottom, and then added 7.5 cm organic soil (moss peat inoculated with fresh sieved peat) to achieve a total depth of 15 cm. In the heterogeneous treatment (T2) we used five different combinations of soil layer depths (mineral/organic soil depth): d1 = 3/12 cm, d2 = 5.25/9.75 cm, d3 = 7.5/7.5 cm, d4 = 9.75/5.25 cm, and d5 = 12/3 cm. We used Irish moss peat (for horticultural use) inoculated with fresh peat collected in a heather moorland at the Glensaugh Research Station, Laurencekirk, UK (56°54′N, 2°34′W), and sieved through a 5 mm mesh, to create the O-horizon. Overall, the same amounts of mineral and organic soil were used in both treatments to prevent differences in nutrient content. In the heterogeneous treatment, specific soil depths were allocated to individual cells such that a particular depth never occurred more than once per row and column within the inner 25 compartments within each replicate. Approximately 150 g litter (dry weight) was placed on top of each replicate initially, and 75 g was added one month later, such that a total of 225 g litter was added to each replicate to maximise potential colonisation. The litter, which consisted mainly of birch leaves and twigs, was collected at the site, and homogenized before being added to the treatments.

We sampled the experiment a year after establishment, in May 2008. From all replicates of both treatments we collected one composite sample at depths of 0–3 cm and one at 3–6 cm depth from each cell in the organic horizon below the litter layer. In each treatment we sampled compartments containing an organic horizon depth of 7.5 cm (d3) only (single depth sample). The composite sample from each depth was composed of 3 sub-samples measuring 5×5 cm and 3 cm depth collected from three of the inner 25 cells in each replicate to avoid any edge effect. From all replicates of the heterogeneous treatment we collected another composite sample at 0–3 cm depth, which contained a single sample from d1, d3 and d5 (mixed depth sample). This pooled sample was collected to determine whether any treatment effect was due to the organisms considering each organic depth (d1, d3, d5) as a unique microhabitat rather than dispersing between cells with these different depths. The samples were placed in plastic tubs and stored at 4°C until processed.

### Soil biota

Extraction of microarthropods commenced the day after the samples were collected. Extractions were performed using modified Tullgren funnels by gradually increasing the temperature to 40°C over 8 days. All individuals of mites from Oribatida and Mesostigmata were counted, and adults (excluding Brachychthonioidea due to difficulties with reliable identification at species level) were identified to species level whenever possible. All individuals of collembolans were counted, and the richness was estimated by sorting through the samples and determining all morphotypes. These were then mounted on slides, and identified to species level where possible. Nematodes were extracted over 48 hrs using a modified tray method version of the Baermann funnel method within 5 days from collecting the samples. After extraction the nematodes were settled into 4 ml vials. The total number of nematodes was estimated by counting the individuals in a known proportion of the sample at 40×magnification. The samples were then mounted on glass slides, and 50 individuals per sample at 0–3 cm and 3–6 cm depth were identified to species level when possible or classified as morpho-species.

The soil samples for microbial analysis were processed within two weeks of collection. The three cores from each sample point were bulked and sieved fresh through a 2 mm mesh. The soil was mixed thoroughly before sub-sampling for microbial analysis. The sub-samples were then stored at −80°C until analysis. The fungal, bacterial and archaeal community composition was analysed using the multiplex-terminal restriction fragment length polymorphism method (M-TRFLP) as described in Singh et al. [42]. We used the primers ITS1 (FAM) and ITS4 for fungi, 63F and 1087R-VIC for bacteria, and Ar344 and Ar927 (NED) for archaea. This analysis produces terminal restriction fragments (TRF) of different lengths, each represented by a peak of varying intensity depending on their abundance. To avoid errors due to data processing we limited the fragments used to those between 50 and 500 base pairs long with a height of 25 relative fluorescent units (rfu) or more, while also excluding peaks with a relative abundance of <0.1%.

### Data analysis

Simpson's Evenness, E_1/D_ [43], of microbial communities was calculated using relative abundances multiplied by 1000 to obtain whole numbers. Differences in the abundance and species richness of the different groups of soil organisms, and E_1/D_ of microbial communities, between treatments in the upper 0–6 cm (0–3 and 3–6 cm depth combined to form one sample) were tested using one-way ANOVAs with group as a random blocking factor. Differences in species richness of the soil organism groups and E_1/D_of microbial communities between d3 and mix samples collected in the heterogeneous treatment were tested for with Student's two-sample paired t-test. Data were log-transformed when necessary. All statistical tests were performed using GenStat version 10.1 (VSN International Ltd., Hemel Hempsted, UK).
